# Improving IoT Cybersecurity Performance with Lifecycle-Motivated Bit-Manipulation Compiler Optimizations

**DOI:** 10.3390/s26113301

**Published:** 2026-05-22

**Authors:** Alexia Budiul, Ciprian Pungilă

**Affiliations:** Department of Digital Technologies and Software Engineering, Faculty of Informatics, West University of Timișoara, 300223 Timișoara, Romania; alexia.budiul04@e-uvt.ro

**Keywords:** IoT security, embedded systems, LLVM, compiler backend, instruction selection, bit manipulation, SHA-256, code-size, cycle-count optimization

## Abstract

Implementing cryptographic primitives on resource-constrained IoT devices involves tight latency, code-size, and energy budgets. This work proposes a general LLVM backend instruction-selection strategy that recognizes single-bit update idioms—typically expressed as LOAD–-(AND/OR)–-STORE sequences in SHA-256 and similar bit-oriented code—and lowers them to the most efficient target-specific bit-manipulation primitive when legality and cost conditions are met. As a concrete instantiation, we implement the strategy for the Renesas RL78/G23 ISA by rewriting eligible patterns into SET1/CLR1 instructions when the constant mask targets exactly one bit. We evaluate the resulting backend on an RL78/G23 platform using cycle counts and code size (bytes) across SHA-256-driven workloads motivated by firmware integrity checking, Merkle-tree hashing, HMAC-based authentication, password-based key derivation (PBKDF2), and chunk-level update validation. The observed cycle reductions are also converted to absolute time across the device’s supported on-chip oscillator frequencies to quantify latency impact under different clocking modes. The experimental validation in this work is limited to the RL78/G23 backend implementation. The underlying instruction-selection idea may be adaptable to other RL78-family devices or to other embedded architectures that provide equivalent single-bit set/clear or bitfield operations; however, such adaptations require target-specific legality checks, cost modeling, and separate experimental validation.

## 1. Introduction

The problem of cybersecurity performance in the world of Internet of Things (IoT) becomes increasingly important in a world where the internet is at risk of a wide range of cyberattacks. This implies the need for strong and complex safety checks to verify data integrity and security, such as validation of firmware integrity, Merkle trees, WiFi pairing with password, TLS handshake for the Web, updates over the air (OTA) for IoT devices, and HMAC-based authentication.

### 1.1. Lifecycle-Motivated Scope

In this work, the term lifecycle-motivated refers to the fact that the evaluated cryptographic workloads are selected from security-relevant stages of an IoT device lifecycle. The proposed compiler optimization itself is not dynamically activated by a runtime lifecycle state; rather, it is a backend instruction-selection optimization whose impact is evaluated across workloads that arise during provisioning, deployment, normal operation, authentication, and maintenance/update phases of IoT devices. This distinction is important because the optimization remains target- and pattern-driven, while the experimental methodology is organized around lifecycle-relevant security activities.

Table 1 summarizes the mapping between the IoT lifecycle stages and the specific workloads analyzed in this study. Concretely, password-based key derivation and WiFi pairing represent provisioning and commissioning workloads; firmware integrity validation represents deployment and secure-boot workloads; HMAC-based authentication and TLS message authentication represent normal operational communication workloads; Merkle-tree validation and OTA update verification represent maintenance and update workloads. These stages are representative of resource-constrained IoT devices, where repeated SHA-256 or HMAC-SHA256 computations affect latency, energy use, and code-size constraints.

### 1.2. Security-Focused Workloads

All of the aforementioned use cases ensure the safety of IoT devices and must perform repeated calls of cryptographic algorithms, in a range of 10 to 8,000,000 and above. Carrying out such calls requires plenty of resources, making it more difficult to integrate a computationally intensive cryptographic algorithm for small embedded systems. For that reason, it is essential to use efficient and reliable algorithms.

Our focus starts with the SHA-256 algorithm. Previous efforts in improving the performance of SHA-256 in this field predominantly follow a hardware-focused approach: redesigning a core or an accelerator from a field programmable gate array (FPGA) to be specialized in executing the algorithm efficiently and fast, or customizing the instruction set architecture (ISA) by adding new, specialized instructions. In contrast, our approach implements a pattern-matching backend optimization in LLVM that preserves the existing ISA (no new instructions are introduced) and changes the compiler to recognize specific LOAD–(AND/OR)–STORE idioms that update exactly one bit. Although this software-only approach provides more modest, incremental gains compared to specialized hardware extensions, it offers the advantage of immediate deployability on existing hardware, without the costs of architectural redesign.

Our implementation and evaluation target the Renesas RL78/G23 by mapping eligible idioms to the existing SET1/CLR1 instructions. Therefore, the empirical results reported in this paper should be interpreted as RL78/G23-specific. The same instruction-selection principle may be applicable to other RL78-family devices that provide SET1/CLR1 and sufficient memory resources for the evaluated workloads. More broadly, adapting the optimization to non-RL78 architectures would require equivalent single-bit set/clear or bitfield instructions, target-specific legality checks, cost modeling, and separate validation. We, therefore, treat cross-architecture evaluation as future work rather than as a demonstrated result of the present study.

Specifically, for this paper’s proposed mechanisms, we use (for the experimental/validation setup) a 16-bit microcontroller from the *Renesas Electronics RL78/G23 Low-Power MCUs Group*, which is based on a CISC architecture and is designed primarily for ultra-low-power, cost-sensitive embedded systems, especially in IoT and consumer/industrial domains. We propose a method to identify two patterns of standard instructions, found in the SHA-256 algorithm, and replace them with one of the MCU-specialized bit-manipulation instructions SET1 and CLR1. These instructions perform either a set or a clear operation on a bit from an address, effectively replacing a specific pattern of load–modify–store instructions. We then perform a thorough benchmark of various security-focused scenarios that IoT devices are commonly involved with, in order to assess the impact on performance and code generation of our pipelined design.

Our aim for this study is to propose a cost-efficient solution to improve the performance of security-focused algorithms commonly used in IoT devices, which makes use of the available resources of the device, rather than changing them.

### 1.3. Limitations

This work evaluates the proposed backend optimization in terms of generated code size and simulated cycle counts. We do not perform direct power or energy measurements on physical RL78/G23 hardware. Therefore, the reported cycle reductions should not be interpreted as measured energy savings. They indicate reduced execution work and shorter execution time under the evaluated workloads, which may have favorable energy implications on duty-cycled or battery-powered devices, but a precise energy analysis would require board-level current measurements or an architecture-specific energy model.

Furthermore, the proposed compiler-only optimization is not intended to outperform dedicated cryptographic hardware accelerators or custom ISA extensions in absolute throughput. Hardware accelerators can provide significantly larger speedups, but they require hardware support, silicon area, design effort, or a different target platform. Our approach addresses a different deployment scenario: already manufactured or resource-constrained microcontrollers where the available ISA must be used as effectively as possible without hardware modification.

## 2. Related Work

There are various papers focused on improving the SHA-256 algorithm, but, to the best of our knowledge, relatively few works focus specifically on backend instruction-selection changes that exploit existing bit-manipulation instructions for SHA-256 without ISA extensions or additional hardware. The papers are mainly split into two categories: one in which the focus is to improve the SHA-256 algorithm, through distinct methods from ours, and one in which the method used modifies the toolchain similar to us, but the focus is not the SHA-256 algorithm; it is the general security improvement of IoT devices, such as detecting vulnerabilities and preventing code reuse attacks.

**Figure 1:**
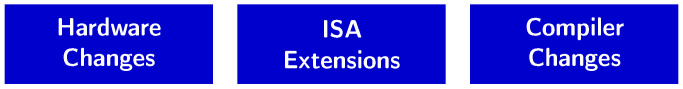
Classification of studied approaches: Hardware Changes [1,2,3,4,5,6,7,8]; ISA Extensions [9,10]; and Compiler Changes [11,12].

After analyzing more than 80 different publications using keywords such as “compile time optimization IoT for cybersecurity” or “SHA-256 embedded systems performance”, we concluded that those in Figure 1 are the most relevant to our study.

The first category of studies—*Hardware Changes*—addresses the challenge of accelerating the SHA-256 algorithm by redesigning the hardware architecture. Wong et al. [1] focused on runtime constraints in IoT and proposed a solution based on two fundamental hardware changes: architectural folding and a 4–2 adder compressor. Similarly, Kulkarni et al. [2] proposed replacing standard carry save adders (CSA) with carry look ahead adders (CLA) and carry skip adders (CSkA), paired with clock gating and power gating techniques, to address power reduction for RFID and IoT devices. Padhi and Chaudhari [3] implemented a pipelined architecture that modifies the compressor and expander block of the hash function, also using a carry skip adder to improve the performance of Xilinx Virtex-4 FPGAs.

Tran et al. [4] concluded that the time it takes for data to travel during the execution of SHA-256 was slowing down the algorithm; therefore, they proposed an accelerator, tested again on an FPGA. Kieu-Do-Nguyen et al. [5] proposed a technology-independent SHA-256 computing core that makes use of the algorithm’s parallelism level and can be used by different FPGA families.

De Barros Santos Júnior [6] focused on blockchain and IoT applications and, similar to Kieu-Do-Nguyen et al., exploited the parallelism of SHA-256, conducting experiments on FPGAs. The former proposed a design with 16 parallel cores, achieved on a Xilinx Virtex-6 FPGA. This study also conducted experiments on other hardware architectures, such as 8-bit and 16-bit microcontrollers, general-purpose processors, and GPUs.

In contrast to these studies, our proposed method does not require any change in the existing hardware; it mainly targets software improvements on already manufactured microchips, thus representing a cost-effective and more reproducible alternative to improve such an intensive bit-manipulation algorithm. Over time, numerous such optimizations could potentially add up and achieve a considerable gain in energy efficiency, extending the device’s battery life and its real-time responsiveness.

Finally, the last remaining papers from this category propose a hardware/software (HW/SW) approach, applied on FPGAs. Issad et al. [7] opted for a solution implemented on a programmable system on chip (PSoC) architecture that executes the SHA-256’s padding operation on the SW side, for flexibility, specifically on a Xilinx Microblaze processor, and computes the intensive arithmetic operations of the algorithm on the HW, to accelerate the execution.

Kammoun et al. [8] conducted a study on two hardware design methods—low-level synthesis (LLS) and high-level synthesis (HLS)—and concluded that LLS presented a significant improvement of 80% in FPGA resources and 15% in throughput, compared to HLS. Therefore, in this specific domain of applicability, the low-level approach outperformed the high-level automated generation, a conclusion that can serve as an argument for our approach, specifically, that low-level optimizations similar to ours are justifiable, and relying solely on automated generation is not always the optimal strategy.

The second category of studies—*ISA Extensions*—also addresses the challenge of accelerating the SHA-256 algorithm, but this time, through a different approach from the first category. De Araujo Gewehr and Moraes [9] conducted a study very similar to ours, namely, they introduced two specialized instructions from the Ibex RISC-V ISA extensions (i.e., Zkne and Zknh) and analyzed the differences they made in area cost and stack usage density for different cryptographic algorithms, such as AES-128, AES-256, SHA-256, and SHA-512. Their study illustrated clock cycle count gains of 42.57 times.

On a similar note to Araujo et al, Sivanesan et al. [10] proposed a detailed instruction-set customization study to accelerate SHA algorithms, on a customizable Tensilica Xtensa core. The instructions they designed resulted in a 30% improvement in performance, compared to implementations that used generic instructions; the paper further highlighted the importance of the SHA-256 hash function for blockchain and cryptocurrency mining.

The impressive results obtained in this category of studies highlight the impact that changes from the early stages of development can have on overall system throughput and energy efficiency.

The last category of studies—*Compiler Changes*—presents recent papers that address the threats embedded IoT systems face today, proposing compiler-level solutions to mitigate these risks at the binary level.

In the context of resource-constrained IoT and embedded devices, Brant et al. [11] addressed the limitations of dynamic information flow tracking (DIFT) techniques, a reliable method for device behavioral analysis, designed for high-performance computing systems. They proposed a lightweight, compiler-based DIFT system called CO-DIFT, suitable for embedded devices, which uses a framework built upon the LLVM compiler infrastructure and integrates security instructions into the executable code, thus preventing control flow attacks. This methodology of manipulating the toolchain is similar to ours, although our paper focuses on architecture-specific optimizations, while their approach represents a machine-agnostic solution.

Bakiris and Oikonomakis [12] addressed a real-world threat in the medical field: the vulnerability of wearable medical devices to cyberattacks, due to their limited computational power and reliance on wireless communication. To mitigate this problem, they proposed the integration of compiler polymorphism techniques to generate structurally unique binaries for each device, a strategy that, similarly to our approach, involves the manipulation of the binary generation process for IoT devices.

The performance gains obtained by the proposed compiler-only optimization are intentionally more modest than those reported by hardware accelerators or ISA-extension-based designs. For example, prior ISA-extension approaches can achieve order-of-magnitude improvements by adding specialized cryptographic instructions or modifying the target architecture. Such results are not directly comparable to the present work, because our method does not introduce new instructions, does not require hardware redesign, and does not assume a different processor implementation. Instead, the proposed optimization exploits bit-manipulation instructions already available in the RL78/G23 ISA. The resulting trade-off is, therefore, between absolute performance and deployment cost: hardware and ISA-extension approaches are preferable when maximum throughput is required and architectural changes are possible, whereas the proposed compiler-backend optimization targets already manufactured or cost-constrained devices where software-only deployability is the main constraint.

To conclude this section, in contrast to these three distinct directions on the topic of IoT cybersecurity that we analyzed, our approach focuses on maximizing the capabilities of existing hardware and instruction set architecture. This solution represents a trade-off prioritizing zero hardware cost over massive performance gains, enhancing the performance of the SHA-256 algorithm and, consequently, of various security mechanisms that rely on this cryptographic algorithm.

## 3. Implementation

### 3.1. Proposed Design Pipeline

This subsection aims to place our study in the standard compilation process used by the LLVM infrastructure, and it is based on LLVM’s compilation workflow as described in *Getting Started with LLVM Core Libraries* [13].

According to their documentation, the following are the three main parts of the LLVM infrastructure:

- **Frontend**: This part is responsible for translating the source code into the compiler’s intermediate representation through lexical, syntactic, and semantic analyses. In our case, as the source code is written in the C programming language, this step is handled by the Clang compiler.
- **LLVM Intermediate Representation (IR)**: It combines human-readable and binary-encoded representations and acts as the middle point between the frontend and the backend.
- **Backend**: This part converts the LLVM IR into object code binaries or target-specific assembly code, through several steps which include the Instruction Selection, Instruction Scheduling, Register Allocation, and Code Emission phases.

**Figure 2:**
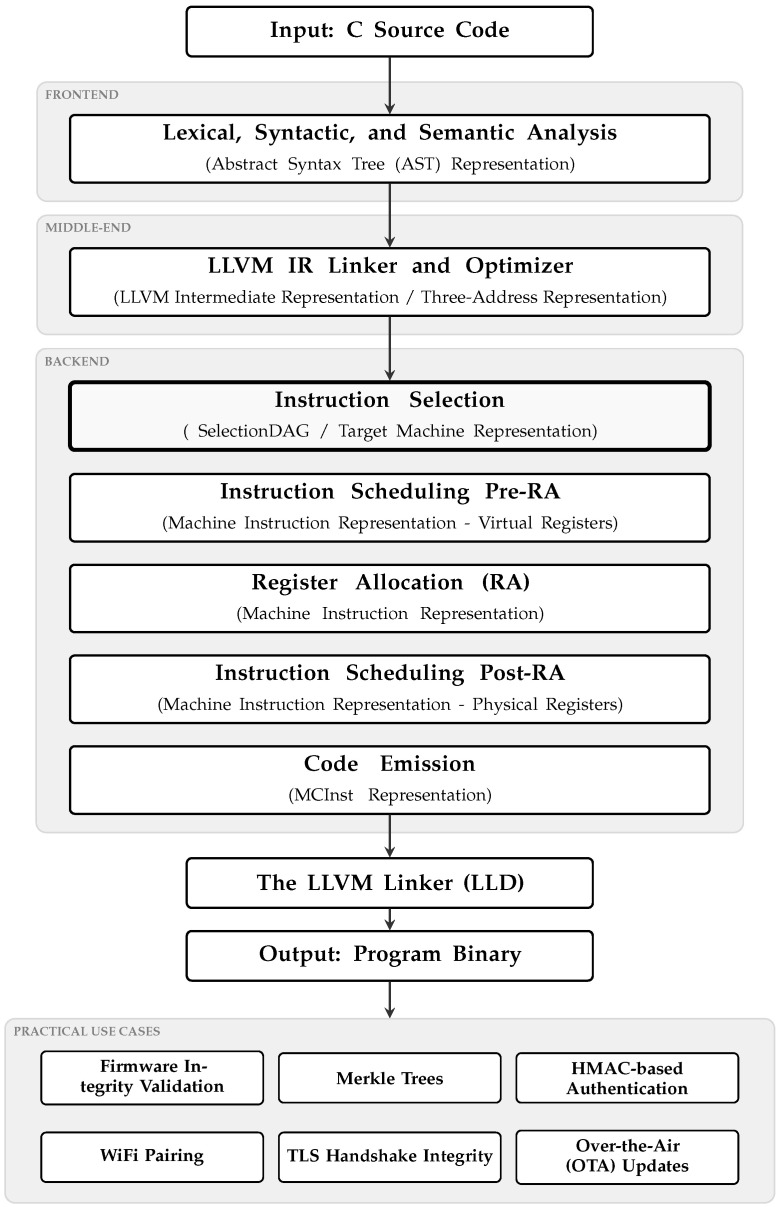
End-to-end compiler workflow: from C source code to IoT deployment using the low-power RL78 MCU.

Our suggested optimization takes place in the Backend, specifically in the Instruction Selection phase. In this stage, the linear LLVM IR is transformed into a directed acyclic graph (DAG) form, a representation that is meant to ease the problem of data flow dependencies among instructions. After this transformation, it follows the execution of tree-based pattern-matching instruction-selection algorithms that are responsible for converting generic LLVM IR nodes into target-specific machine nodes. Our approach arises within this step, targeting the function MatchSET1CLR1, which introduces the bit manipulation instructions SET1 and CLR1. The end-to-end workflow, transitioning from C source code to practical SHA-256 use cases in IoT, is illustrated in Figure 2.

### 3.2. Target Architecture

As mentioned in Section 1, our approach targets the 16-bit Renesas RL78/G23 microcontroller group [14]. From an architectural perspective, RL78 follows a Complex Instruction Set Architecture, which prioritizes complex instructions that reduce memory usage for low-power microcontrollers. For that reason, the ISA contains instructions that are faster and more efficient than the standard general-purpose ones and represent great candidates for bit-intensive cryptographic algorithms, such as the SHA-256 hash function.

### 3.3. Technical Details

This subsection aims to describe the algorithm behind the aforementioned MatchSET1CLR1 function, which replaces a pattern containing three generic LLVM DAG nodes with a single, target-specific node. The correctness of this backend optimization was verified through the execution of a full regression test suite containing a total of 65,545 tests, all of which resulted in no unexpected failures, thus confirming that the optimization does not compromise the toolchain’s integrity.

**Figure 3:**
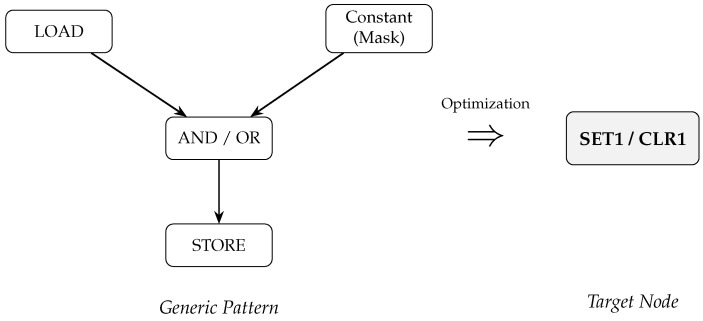
Visual representation of the instruction-selection optimization: transforming a generic read–modify–write pattern into a specialized bit-manipulation instruction.

Please refer to Figure 3 for a visual representation of this algorithm.

This process takes place in the Instruction Selection phase, as already mentioned, and can be broken down into three stages: Pattern Recognition, Safety Constraints Validation, and Instruction Substitution.

1. Pattern Recognition
  The function is invoked when a generic STORE node is encountered. In that case, as the SelectionDAG represents data dependencies through its tree-like structure, it traverses the graph upwards and verifies that the STORE node is part of one of the following sequences: LOAD–AND–STORE, which could potentially represent a bit-clear operation, or LOAD–OR–STORE, which could potentially represent a bit-set operation.
2. Safety Constraints Validation
  Several aspects are verified within this stage to confirm that the pattern-matching conditions are met, but only the most relevant ones are mentioned below:
  First, the algorithm checks that the machine value type (MVT) of the logical operation node targets 8-bit or 16-bit integers, and that the LOAD and STORE nodes use the same address, enforcing memory consistency.
  Furthermore, the logical operation node must have only one use within the basic block to avoid side effects, and its second operand must be a constant value. Moreover, to ensure that the pattern modifies a single bit from the address in memory, the constant value (also called the mask) must be validated accordingly:
  - For the logical OR operator, the constant mask must be a single-bit value (i.e., exactly one bit set) in the operand bit-width;
  - For the logical AND operator, the constant mask must be an all-ones value except for exactly one cleared bit in the operand bit-width (equivalently, the bit-width-truncated complement of the mask is a single-bit value).
3. Instruction Substitution
  After all safety constraints are fulfilled, the final phase computes the target bit index as the position of the single set bit. Concretely, for a power-of-two mask *m*, the bit index is ctz(m) (count trailing zeros), which equals $\left\lfloor \log_{2}\left(m\right)\right\rfloor$ in integer arithmetic; no floating-point logarithms are used.
  At this point, a specific adaptation is required for 16-bit operations (i.e., MVT::i16), as the SET1 and CLR1 instructions operate at byte level. If the target bit resides in the upper part of the 16-bit value, at indices 8–15, then the algorithm does the following:
  - Address adjustment: The memory address is incremented by an offset of 1 byte.
  - Mask normalization: The mask used is right-shifted by 8 positions, to transform it into an 8-bit immediate.
  After these changes, the operation is effectively treated as a regular 8-bit manipulation.
  Finally, the generic DAG nodes are replaced by the new target-specific node—either SET1 or CLR1—configured with the computed bit_index and the corresponding address, according to the architecture’s syntax:
                  SET1 addr.bit_index
                  CLR1 addr.bit_index

Through the MatchSET1CLR1 function, the compiler is now able to make use of the unique capabilities of the CISC instructions, an optimization that would not have been possible otherwise. Specifically, we managed to replace three instructions with a single one, thus eliminating two instructions from the final code. While this may appear negligible for a single replacement, considering that the SHA-256 algorithm performs intensive bit operations and that specific applications could invoke this algorithm over 5,000,000 times, the cumulative improvements are considerable. Furthermore, if multiple similar optimizations were performed, the combined results could become substantial.

Ultimately, by reducing the size of the final code, we reduce the memory usage and execution time, aspects that are crucial for low-powered devices such as our targeted microcontroller.

#### Trigger Count in the Evaluated SHA-256 Implementation

In the SHA-256 implementation evaluated in this work, the proposed SET1/CLR1 transformation is triggered during the SHA-256 initialization phase, not inside the 64 compression rounds of the SHA-256 compression function. Therefore, the number of optimized SET1/CLR1 replacements per compression round is zero. Dynamically, each SHA-256 computation executes one optimized state-bit update sequence corresponding to the matched load–logical operation–store idiom.

This explains the fixed cycle saving observed across the evaluated workloads. In the relevant RL78/G23 addressing form, both SET1 and CLR1 require 2 cycles. The original instruction sequence requires 9 cycles in the evaluated case, whereas the optimized sequence requires 4 cycles, producing a net saving of 5 cycles per SHA-256 computation. As the evaluated workloads repeatedly invoke the same SHA-256 routine, the total cycle saving scales with the number of SHA-256 calls.

The number of static SET1/CLR1 mnemonics visible in the generated assembly may differ across benchmark programs because of inlining and because some workloads instantiate or invoke SHA-256 multiple times. For example, HMAC-based workloads involve two SHA-256 computations per HMAC operation. These static assembly occurrences should, therefore, not be interpreted as additional replacements inside the SHA-256 compression loop; the dynamic optimization effect remains tied to the initialization phase of each SHA-256 computation.

### 3.4. Algorithm Description

Throughout this paper, the SHA-256 algorithm has been mentioned multiple times. This subsection aims to describe its behavior, importance, and application in the use cases that represent the object of our experiments.

*Secure Hash Algorithm 256-bit (SHA-256)* is a cryptographic function that produces a unique 32-byte (256 bits) hash for the provided input, through complex rounds of bitwise operations (e.g., AND, XOR, rotations, shifts) and specific constants and functions. Due to its heavy reliance on bit manipulation operations, the algorithm represents a suitable candidate to validate the impact of the proposed instructions SET1 and CLR1. To verify cryptographic correctness, both the baseline and optimized SHA-256 binaries were validated against standard NIST [15] SHA-256 test vectors from FIPS 180-4. In all tested cases, the generated digests matched the expected reference outputs, confirming that the backend transformation preserves the functional behavior of the SHA-256 implementation.

Considering its security and performance characteristics, the SHA-256 algorithm is integrated into numerous security protocols and everyday cybersecurity contexts.

**Figure 4:**
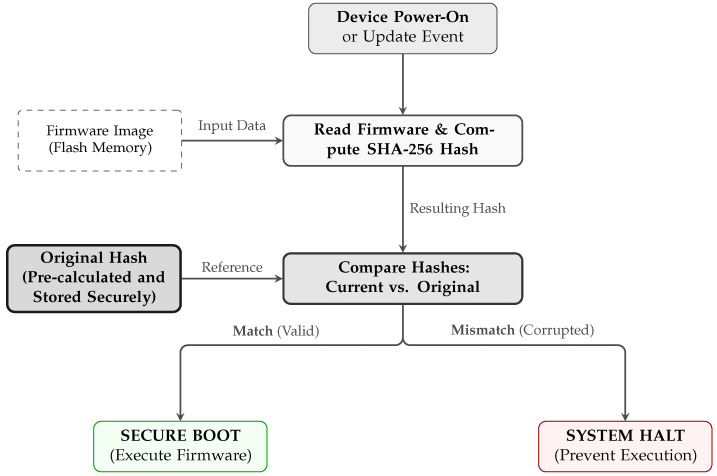
Secure boot decision logic for firmware integrity validation.

*Firmware Integrity Validation* is a security procedure applied when a device is turned on or when it receives an update, and its purpose is to verify that the source code of a device has not been corrupted. Before the release, the source code is compiled—representing the firmware image—and then passed through the SHA-256 algorithm, generating a unique hash. When the device powers on, the source code is read from memory and the current hash is computed, then it is compared with the original one. If the two are identical, the firmware is valid and the system boots; otherwise, the boot is halted to prevent the execution of compromised code. For a graphical representation of this process, refer to Figure 4.

*Merkle Trees* represent a hierarchical approach for efficiently verifying large datasets in IoT devices, by decomposing the data into smaller pieces (i.e., leaf nodes and non-leaf nodes) and allowing each of them to be verified independently. They are also heavily used in various popular blockchains, such as Bitcoin. Functionally, every leaf node is labeled with the cryptographic hash of a data block, and every non-leaf node is labeled with the cryptographic hash of the concatenated hashes of its child nodes. The SHA-256 algorithm is used to compute the hashes of all the nodes, and it is applied recursively from the leaves up to the root—which represents the fingerprint of the entire dataset. If one of the leaves is corrupted, the root’s hash will change as well, and the precise location of the change will be easily identifiable by traversing the tree downwards to the specific corrupted node.

**Figure 5:**
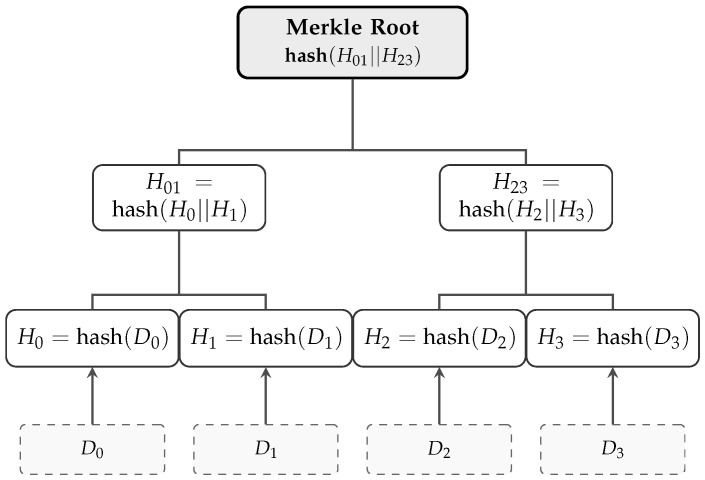
Hierarchical structure of a Merkle tree.

As illustrated in Figure 5, the verification process is structured into several layers:

- Data Blocks ($D_{0}$ to $D_{3}$): These represent the raw datasets or the memory segments partitioned for independent verification;
- Leaf Nodes ($H_{0}$ to $H_{3}$): These are the outputs of the SHA-256 algorithm applied to each data block $D_{i}$;
- Non-leaf Nodes ($H_{01},H_{23}$): These are generated by concatenating the hashes of their child nodes and re-applying the SHA-256 algorithm to the combined result (e.g., $H_{01}=\mathrm{hash}\left(H_{0}\|H_{1}\right)$);
- Merkle Root: This serves as the final hash at the top of the hierarchy and acts as a unique cryptographic fingerprint for the entire dataset.

Any corruption in a specific block $D_{i}$ causes a mismatch in its corresponding hash $H_{i}$, which propagates through the intermediate nodes to the Merkle root, allowing the system to rapidly identify and isolate the corrupted data block.

To illustrate the practical utility of Merkle trees in resource-constrained environments, consider a scenario involving an RL78-based field device (e.g., a smart meter, a thermostat, an industrial sensor) that receives firmware updates over bandwidth-limited links like BLE, LoRaWAN, narrowband cellular, or RS-485 via gateways. In these scenarios, the RL78 microcontroller lacks sufficient RAM and cannot buffer the entire firmware image in RAM; thus, the firmware image is split into fixed-size chunks.

During the update, the device receives the chunks along with its corresponding Merkle inclusion proof, which consists of their siblings’ hashes and the positional bits required for path reconstruction. The process follows the steps below and is illustrated in Algorithm 1.

- The SHA-256 algorithm is applied to each received chunk to obtain its corresponding leaf hash;
- The directional bits are used to compute parent hashes by concatenating the current hash with its sibling in the order specified by the inclusion proof;
- This process is continued until a candidate root is generated, which is compared against a pre-verified trusted hash root;
- If the hashes match, the chunk is committed to flash memory; otherwise, the update is rejected.

**Algorithm 1** Merkle path verification for RL78 updates.
**Require:** 
Trusted Root $R_{trust}$, Chunk $C_{i}$, Sibling hashes $\left\{S_{0},\ldots ,S_{n}\right\}$, Direction bits *D*1:

$H_{curr}\leftarrow \mathrm{SHA}-256\left(C_{i}\right)$

2:**for** *j* from 0 to *n* **do**                      ▹ Iteratively reconstruct the path up to the root3:    **if** bit *j* of *D* indicates $H_{curr}$ is Left **then**4:        $H_{curr}\leftarrow \mathrm{SHA}-256\left(H_{curr}\|S_{j}\right)$5:    **else**6:        $H_{curr}\leftarrow \mathrm{SHA}-256\left(S_{j}\|H_{curr}\right)$7:    **end if**8:
**end for**
9:

$R_{candidate}\leftarrow H_{curr}$

10:**if** $R_{candidate}==R_{trust}$ **then**11:    **Commit** $C_{i}$ to flash memory12:
**else**
13:    **Reject** $C_{i}$14:
**end if**



**Figure 6:**
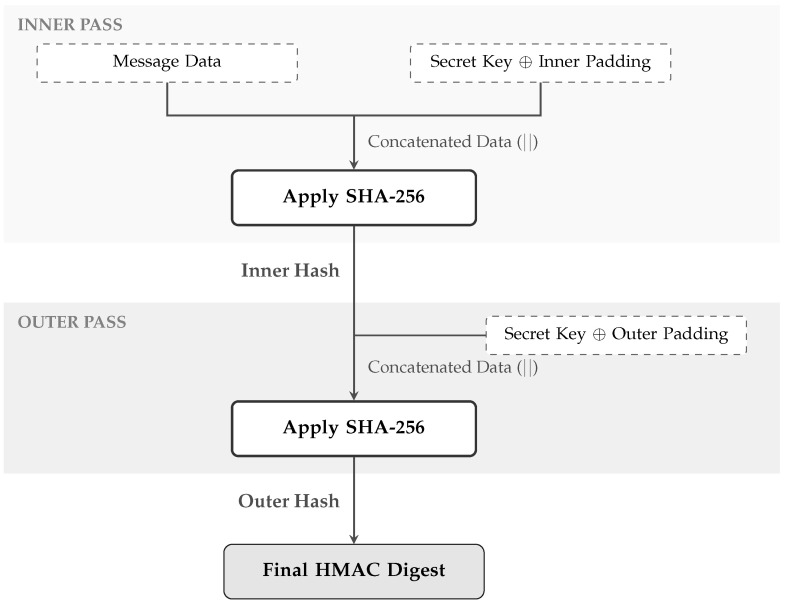
HMAC-SHA256 architectural flow.

The *Hash-Based Message Authentication Code (HMAC)* represents a building block for security protocols designed for network authentication and secure web communications. The algorithm behind it is a combination of SHA-256—which ensures data integrity—and a shared secret key that certifies the authenticity of the data. Structurally, it involves nested computations of SHA-256, specifically: the inner hash is derived by applying the algorithm on the data transmitted and the secret key (combined with an inner padding), and the outer hash is derived by applying the algorithm on the inner hash obtained and the secret key (combined with an outer padding). The final result is the HMAC Digest, a cryptographic seal for data authentication, which follows the execution flow illustrated in Figure 6.

*WiFi Pairing with Password* represents one of the most computationally intensive use cases of HMAC-based authentication and aims to avoid brute force attacks when connecting to a wireless network. This process is also known as Wifi Protected Access (WPA2), and it works as follows: the user’s password is combined with the network’s name (i.e., the SSID)—a process called salting and performed by a key derivation function called PBKDF2 (Password-Based Key Derivation Function 2). The result is passed to the HMAC algorithm 4096 times [16,17] to derive the final session key. This process is meant to be slow to achieve high security.

The *Transport Layer Security (TLS) Handshake* represents an indispensable process for establishing a secure web session, critical to the integrity of the Internet [18]. This protocol is executed by a web browser each time an HTTPS website is accessed on the Internet, but it is equally important in consumer IoT products and non-browser IoT applications; more specifically, it allows a safe communication between the central server and the sensor, which uses the MQ Telemetry Transport (MQTT) protocol to transmit relevant data [19]. Similar to WiFi pairing, the HMAC algorithm is used to validate the integrity of all messages exchanged between the device and the server. After the two exchange several security-related parameters, just before establishing a secure connection, the HMAC algorithm is applied with a secret key both parties know, thus ensuring the authenticity and integrity of the conversation.

**Figure 7:**
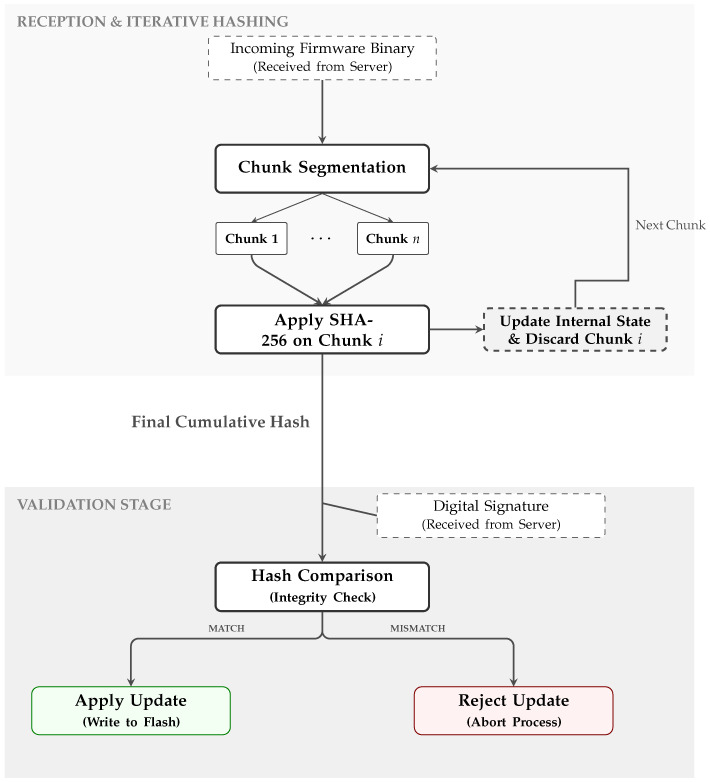
Iterative SHA-256 workflow for OTA updates.

The last use case discussed in the context of SHA-256 represents *Updates Over the Air (OTA)* for IoT devices. As highlighted in [20], updating the software or firmware running on an IoT device ensures the correction of bugs—particularly security bugs—and the addition of new features. Moreover, this use case ensures safe updates remotely, an aspect that is critical for devices located in less accessible areas. Briefly, the process of an OTA update is the following: the incoming update is processed into smaller chunks, on which the SHA-256 algorithm is applied iteratively to update the internal state. After the final chunk is processed, the resulting cumulative hash is compared against the digital signature received from the server. If they are equal, the update is written in memory. In case of a mismatch between the two, the update is rejected and does not affect the functionality of the device. Figure 7 exemplifies this process.

### 3.5. Experimental Configuration and Workload Analysis

Following the theoretical descriptions of the algorithms, this subsection outlines specific implementation details and analyzes the workload in terms of required SHA-256 calls for each use case.

The experimental configuration for RL78/G23 was established using the LLVM for Renesas RL78 17.0.1.202506 toolchain version [21], together with a binary size utility llvm-size (version 17.0.1) and an instruction set simulator rl78-elf-sim (a component of the GNU Debugger, version 16.2).

A benchmark analysis was performed for each test case in two distinct build states of the Clang compiler: a baseline version and an optimized version, incorporating the MatchSET1CLR1 backend logic.

The workflow applied was structured as follows:

- For the execution speed analysis:
  This analysis aims to quantify the reduction in CPU clock cycles, and conforms to these steps:
  1. Both compiler versions were used to generate binaries using the -O3 flag, which enables an aggressive optimization for speed, required in this specific case to better highlight the speed difference.
  2. The resulting binaries were executed through the rl78-elf-sim simulator to obtain the total number of CPU clock cycles.
- For the code size analysis:
  This analysis quantifies the reduction in different memory segments (e.g., the .text area) and conforms to these steps:
  1. Similarly to the execution speed analysis, the two compiler versions were used to generate binaries by applying the specific flag -Oz, in this case one that prioritizes the code size above the other metrics.
  2. The llvm-size utility was used to extract the byte count from the resulting binaries, across different memory segments.
  In both analyses, the primary -O3 and -Oz flags were paired with some other auxiliary flags, essential for the benchmark process.

To ensure measurement precision, the final results obtained in Section 4 were isolated from the overhead of buffer initialization, data generation, function calls, and loop logic by creating two source files for each scenario: a full implementation and an overhead-only version. This differential approach allowed for the extraction of the net execution time by subtracting the cycles or bytes consumed by non-cryptographic operations from the total count. Regarding memory placement, the implementation of the various use cases allocated the workload data (i.e., the input vectors and dynamic buffers) in RAM.

Each experiment used randomly generated input data, but, given the hardware limitations of the RL78 microcontroller, the experiments with a higher workload were performed using techniques such as buffer reuse, for splitting the large amount of input data. (e.g., firmware integrity validation, Merkle trees, and OTA updates).

- Firmware Integrity Validation: The experiments were performed on a 512 KB and 768 KB firmware image, which was processed in chunks of 2 KB, 4 KB and 8 KB, resulting in the following number of SHA-256 calls:
  – For a 512 KB image: 256 calls (2 KB chunks), 128 calls (4 KB chunks), and 64 calls (8 KB chunks);
  – For a 768 KB image: 384 calls (2 KB chunks), 192 calls (4 KB chunks), and 96 calls (8 KB chunks).
- Merkle Trees: The experiments were performed directly on 1024 leaves (i.e., $2^{10}$), approximately 1 million leaves (i.e., $2^{20}=1,048,576$), 2 million leaves (i.e., $2^{21}=2,097,152$), 4 million leaves (i.e., $2^{22}=4,194,304$), and 8 million leaves (i.e., $2^{23}=8,388,608$). The larger configurations were executed using the same fixed-buffer reuse methodology used for the smaller configurations, so the benchmark does not require the entire Merkle tree or the full dataset to reside in RAM at once. As every full Merkle tree has 2N−1 nodes, where *N* is the number of leaves, the corresponding SHA-256 calls are the following:
  – For 1024 leaves: 2047 calls;
  – For 1M leaves: 2,097,151 calls;
  – For 2M leaves: 4,194,303 calls;
  – For 4M leaves: 8,388,607 calls;
  – For 8M leaves: 16,777,215 calls.
- HMAC-based Authentication: The experiment simulated a 24-h continuous operation cycle, with 1 authentication per second; thus, two SHA-256 calls per second.
  – Workload: 172,800 SHA-256 calls (2 × 86,400 s/day).
- Password-Based Key Derivation (PBKDF2): This experiment models a PBKDF2-HMAC workload with 4096 HMAC iterations, a commonly used iteration count in practice. As each HMAC evaluation involves two SHA-256 computations, the workload is
  – Workload: 8192 SHA-256 calls.
- TLS Message Authentication (HMAC-based suites): This experiment models an exchange of 30 authenticated messages/records protected with HMAC (note that the exact count depends on the TLS version and cipher suite). Consequently:
  – Workload: 60 SHA-256 calls.
- Chunk-Level Update Validation: In this experiment, we model a chunk-based integrity scheme in which a 1 MB update is validated by hashing each received 512-byte chunk independently (as opposed to streaming a single hash over the entire image). This results in
  – Workload: 2048 SHA-256 calls.

## 4. Experimental Results

This section reveals the results obtained from simulating the previously introduced use cases of SHA-256. The metrics evaluated during the experiments are the execution speed (measured in CPU clocks or cycles) and the code size (measured in bytes).

Considering the on-chip oscillator frequencies of the RL78/G23 Group, we calculated the actual time saved for each of the scenarios, measured in seconds (s) or microseconds (µs), with the results illustrated in the tables detailing the time saved.

RL78/G23 specific oscillator frequencies:

- High-speed: up to 32 MHz;
- Middle-speed: up to 4 MHz;
- Low-speed: 32.768 kHz.

Based on these values, we calculated the actual time saved using the following formula:

(1) $$\mathrm{Time}\;\mathrm{Saved}\;\left(s\right)=\frac{\mathrm{Cycles}\;\mathrm{Saved}}{\mathrm{Frequency}\;\left(\mathrm{Hz}\right)}.$$

Table 2 presents the execution speed results obtained from the firmware integrity validation experiment, covering all six cases mentioned in Section 3.5. The column *Cycles Saved* indicates the reduction in CPU clock cycles after applying the compiler optimization, while the following columns under *Time Saved* illustrate the time saved for each of the frequencies mentioned above.

**Figure 8:**
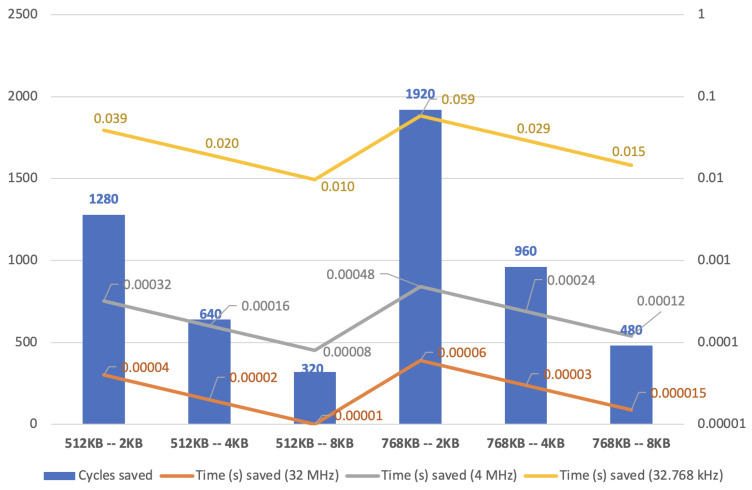
Dual-axis representation of cycles and time saved for firmware integrity validation.

For a clearer understanding of these variations, Figure 8 illustrates the performance impact across the evaluated firmware and chunk configurations.

Table 3 reports the measured speed-related experiments for Merkle trees. It lists the number of leaves, the total number of SHA-256 calls, and the CPU cycles saved for each evaluated configuration.

The measured Merkle-tree results follow the expected linear relation because the workload is dominated by the number of SHA-256 invocations. For a full Merkle tree with *N* leaves, exactly 2N−1 hashes are computed, as each leaf and each internal node is hashed once. In the evaluated implementation, the proposed backend transformation saves five cycles per SHA-256 computation. Therefore, the measured cycle saving for each Merkle-tree configuration is

ΔC=5×(2N−1).

**Figure 9:**
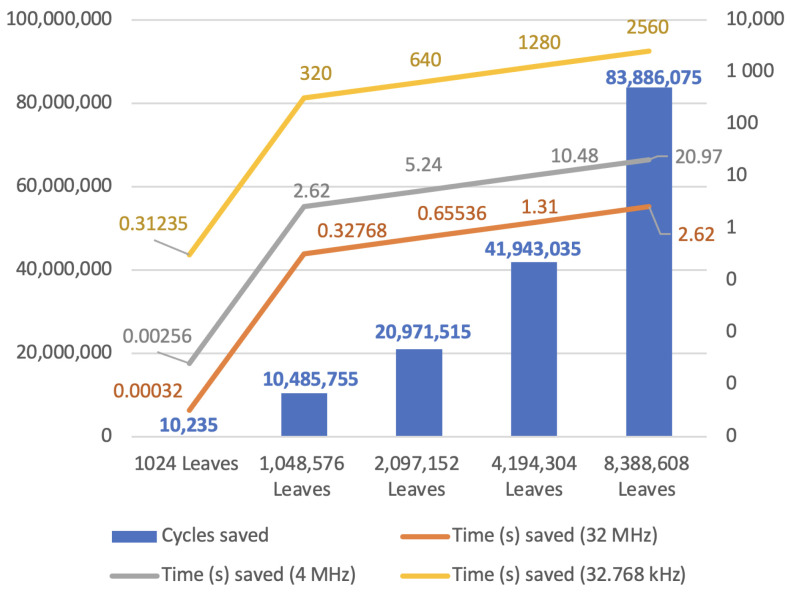
Dual-axis representation of cycles and time saved for Merkle tree scaling analysis.

The agreement between the measured values and this expression confirms that the fixed-buffer reuse methodology remains valid for the larger 2M, 4M, and 8M configurations evaluated in this work. To better understand the scalability of these gains, Figure 9 depicts this linear relationship between the leaf count and the resulting cycle savings.

**Figure 10:**
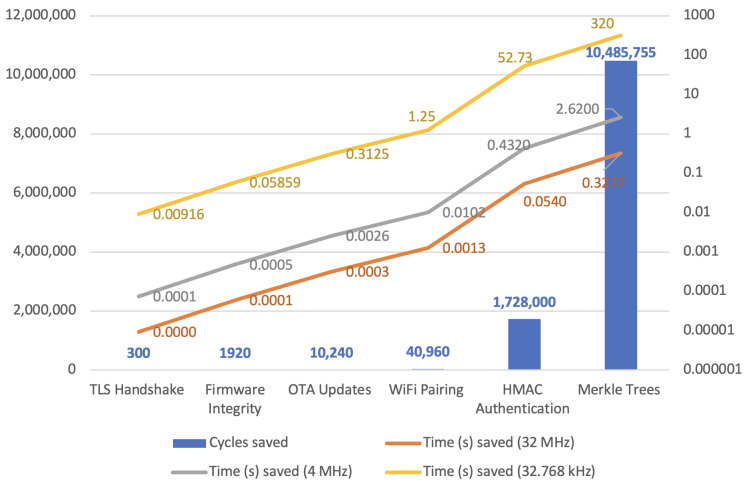
Dual-axis representation of cycles saved and execution time across different frequencies.

Table 4 illustrates the cycles and time saved, choosing a representative scenario for each simulated use case. For firmware integrity validation, a 768 KB firmware size, processed in 2 KB chunks was chosen, and for Merkle trees, a total of 1M leaves was used. Complementing these results, Figure 10 provides a visual representation of the time saved (s) across the three on-chip frequencies, confirming the predictable scaling of the optimization.

Regarding the code size experiments, an interesting conclusion that we draw is that for all the scenarios tested, the difference between the case that used the compiler’s optimization and the one that did not was always consistent. Consequently, our optimization proved to be useful in a specific location of the SHA-256 algorithm, which remains invariant across all use cases.

For the exemplified results of different memory sections, refer to Table 5. The *Difference* column reveals a reduction of 8 bytes in the text area, thus supporting the conclusion that the optimization is independent of the use case implemented. The dec section represents the cumulative reduction across text, data, and bss sections, measured in the decimal system. Similarly, the hex section illustrates the cumulative reduction in the hexadecimal system. In this specific case, the reduction is entirely attributed to the text section.

The 8-byte reduction reported in Table 5 is modest relative to the size of a complete firmware image and should not be interpreted as a substantial global firmware-size reduction. Rather, it reflects the local code-size effect of a narrowly targeted instruction-selection transformation. The primary benefit of the proposed optimization is the reduction in executed cycles across repeated cryptographic workloads, while code-size reduction is a secondary effect. More substantial firmware-size reductions would likely require complementary whole-program techniques, such as link-time optimization, dead-code elimination, function- and data-section garbage collection, cryptographic library specialization, or additional backend peephole optimizations targeting a broader set of instruction idioms. These approaches are orthogonal to the proposed SET1/CLR1 transformation and may be combined with it in future work.

Table 6 brings together all experiments performed on the RL78/G23 microcontroller from Renesas Electronics, and concludes the results regarding the number of clock cycles and the time saved, specified for all three oscillator frequencies of the device.

**Figure 11:**
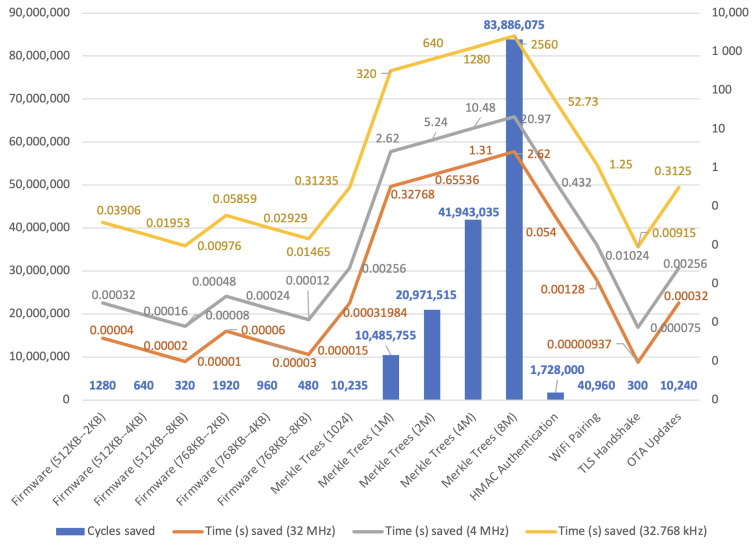
Dual-axis representation of cycles and time saved for evaluated security use cases.

The overall distribution of these execution-time savings across the supported clock frequencies is further synthesized in Figure 11.

Looking at any experiment from Table 6, it can be observed that while the *Cycles Saved* column remains constant, the *Time Saved* column is inversely proportional to the frequency. More precisely, as frequency increases, the time saved decreases. This conclusion can also be drawn by looking at Formula 1, where the time saved is calculated by dividing the number of cycles saved by the frequency used; as frequency increases, the absolute time saved decreases for a fixed cycle reduction. However, the relative speedup (percentage) implied by a given cycle saving is frequency-independent; the frequency scaling here is shown to quantify absolute latency impact under different clocking modes.

The cycle reduction produced by the proposed optimization is independent of the selected operating frequency, but the corresponding absolute time saving depends on the clock frequency. In the RL78/G23 setting considered in this work, 32 MHz represents a high-speed active execution mode suitable for compute-intensive hashing, 4 MHz represents a middle-speed operating point, and 32.768 kHz represents a low-speed clocking mode included to quantify the latency impact under ultra-low-power operation. Therefore, the *32 MHz* and *4 MHz* columns are the most representative for active cryptographic processing, while the *32.768 kHz* column illustrates the upper bound of absolute latency savings under low-speed execution.

This architectural variety of on-chip frequencies exists because each of them has specific advantages and disadvantages [22]:

- High-power mode (32 MHz): This results in a higher energy consumption, but implies a fast execution and better performance. Furthermore, it reduces the actual execution time (AET) and enables the CPU to enter a low-power consumption state faster [23].
- Low-power mode (32.768 kHz): While the performance is lower for this clocking mode, it represents an ultra-low-power operating point. The proposed optimization shortens the execution time in this mode as well; however, this result should be interpreted as an execution-time reduction rather than as a measured battery-power saving.

After an empirical analysis of the impact that the proposed SHA-256 optimizations have on the presented real-world use cases, it can be concluded that the results obtained are favorable for the real-time responsiveness and the code size of the targeted 16-bit CISC architectures. The highest absolute latency reductions at 32.768 kHz range from 5.3 min to 42.6 min. These values should be interpreted as execution-time savings under low-speed clocking rather than as measured energy savings. Although shorter active execution time may be beneficial for duty-cycled or battery-powered deployments, quantifying the actual energy impact would require direct current measurements or a calibrated architecture-specific energy model.

### Scope of Cryptographic Evaluation

The experimental validation in this work focuses on SHA-256-driven workloads. This choice is intentional: SHA-256 is widely used in IoT security mechanisms and exposes bit-oriented code patterns suitable for evaluating the proposed SET1/CLR1 instruction-selection optimization. The proposed transformation is not a general-purpose cryptographic accelerator; it applies only when the compiler identifies eligible LOAD–(AND/OR)–STORE single-bit update idioms. Other algorithms, such as AES and ECC, have different dominant computational structures and may require different backend optimizations. Evaluating whether AES, ECC, or other cryptographic primitives expose similar target-specific optimization opportunities is, therefore, left as future work.

## 5. Conclusions

This paper presents a focused compiler-backend optimization for improving the execution efficiency of SHA-256-driven security workloads on resource-constrained IoT devices. The proposed method targets a specific instruction-selection opportunity in the LLVM backend: the replacement of eligible load–logical operation–store sequences that modify a single bit with the target-specific SET1 and CLR1 instructions available on the Renesas RL78/G23 architecture. By exploiting existing ISA features rather than introducing new hardware support or custom instructions, the approach provides a software-only optimization path for already manufactured or cost-constrained embedded devices.

The experimental evaluation demonstrated that this transformation reduces the number of executed cycles across several SHA-256-based workloads relevant to IoT security, including firmware integrity validation, Merkle-tree verification, HMAC-based authentication, PBKDF2/WiFi pairing, TLS message authentication, and OTA update validation. These workloads were selected because they correspond to representative security activities across the lifecycle of an IoT device, from provisioning and deployment to normal operation and maintenance/update. In this sense, the lifecycle aspect of the work refers to the organization and motivation of the evaluated security workloads, not to a compiler pass that dynamically adapts to the runtime lifecycle state of the device.

The obtained results show that even a narrow backend transformation can provide measurable execution-time benefits when applied to cryptographic operations that are invoked repeatedly. The largest gains appear in workloads with a high number of SHA-256 calls, such as Merkle-tree validation and long-running HMAC-based authentication. The code-size reduction observed in the evaluated implementation is modest, amounting to an 8-byte reduction in the .text section. Therefore, this result should not be interpreted as a substantial reduction of the overall firmware image. Rather, it confirms that the transformation has a small but measurable local effect on generated code size, while the primary benefit of the proposed optimization remains cycle-count reduction.

Overall, this work demonstrates that small, target-aware compiler-backend optimizations can contribute to improving the efficiency of security-critical IoT workloads without requiring hardware redesign. While the individual transformation studied here is intentionally narrow, its results support the broader idea that cumulative compiler-level improvements can help resource-constrained devices execute essential cybersecurity functions more efficiently while preserving compatibility with existing hardware.

## Figures and Tables

**Table 1:** Mapping between IoT lifecycle stages and evaluated security workloads.

IoT Lifecycle Stage	Security Activity	Workload Evaluated in This Paper
Provisioning/commissioning	Password-derived credentials and pairing	PBKDF2/WiFi pairing
Deployment/boot	Firmware authenticity and integrity checking	Firmware integrity validation
Normal operation	Authenticated device communication	HMAC-based authentication
Secure communication	Session/message authentication	TLS message authentication
Maintenance/update	Chunk-level validation and update verification	Merkle-tree validation and OTA updates

**Table 2:** Experimental results for firmware integrity validation (speed and time analysis).

Firmware Size	Chunk Size	Cycles Saved	Time Saved		
			**32 MHz**	**4 MHz**	**32.768 kHz**
512 KB	2 KB	1280	40.00 µs	320.00 µs	39.06 ms
	4 KB	640	20.00 µs	160.00 µs	19.53 ms
	8 KB	320	10.00 µs	80.00 µs	9.77 ms
768 KB	2 KB	1920	60.00 µs	480.00 µs	58.59 ms
	4 KB	960	30.00 µs	240.00 µs	29.30 ms
	8 KB	480	15.00 µs	120.00 µs	14.65 ms

**Table 3:** Measured results for Merkle trees (speed analysis).

Number of Leaves	Total SHA-256 Calls	Cycles Saved
1024 ($2^{10}$)	2047	10,235
1,048,576 ($2^{20}$)	2,097,151	10,485,755
2,097,152 ($2^{21}$)	4,194,303	20,971,515
4,194,304 ($2^{22}$)	8,388,607	41,943,035
8,388,608 ($2^{23}$)	16,777,215	83,886,075

**Table 4:** Consolidated experimental results for all use cases (speed and time analysis).

Experiment	Cycles Saved	Time Saved		
		**32 MHz**	**4 MHz**	**32.768 kHz**
Firmware Integrity	1920	60.00 µs	480.00 µs	58.59 ms
Merkle Trees	10,485,755	327.68 ms	2.62 s	320.00 s
HMAC Authentication	1,728,000	54.00 ms	432.00 ms	52.73 s
WiFi Pairing	40,960	1.28 ms	10.24 ms	1.25 s
TLS Handshake	300	9.38 µs	75.00 µs	9.16 ms
OTA Updates	10,240	320.00 µs	2.56 ms	312.50 ms

**Table 5:** Resulted size difference (measured in bytes).

Section	Description	Difference
text	Code Segment	8
data	Initialized Data	0
bss	Uninitialized Data	0
dec	Total Reduction (Decimal)	8
hex	Total Reduction (Hex)	8

**Table 6:** Time savings based on RL78 operating frequencies.

Experiment	Cycles Saved	Time Saved 32 MHz	Time Saved 4 MHz	Time Saved 32.768 kHz
Firmware Integrity (512 KB–2 KB)	1280	40.00 µs	320.00 µs	39.06 ms
Firmware Integrity (512 KB–4 KB)	640	20.00 µs	160.00 µs	19.53 ms
Firmware Integrity (512 KB–8 KB)	320	10.00 µs	80.00 µs	9.76 ms
Firmware Integrity (768 KB–2 KB)	1920	60.00 µs	480.00 µs	58.59 ms
Firmware Integrity (768 KB–4 KB)	960	30.00 µs	240.00 µs	29.29 ms
Firmware Integrity (768 KB–8 KB)	480	15.00 µs	120.00 µs	14.65 ms
Merkle Trees (1024)	10,235	319.84 µs	2.56 ms	312.35 ms
Merkle Trees (1M)	10,485,755	327.68 ms	2.62 s	320.00 s (5.3 min)
Merkle Trees (2M)	20,971,515	655.36 ms	5.24 s	640.00 s (10.6 min)
Merkle Trees (4M)	41,943,035	1.31 s	10.48 s	1280 s (21.3 min)
Merkle Trees (8M)	83,886,075	2.62 s	20.97 s	2560 s (42.6 min)
HMAC Authentication	1,728,000	54.00 ms	432.00 ms	52.73 s
WiFi Pairing	40,960	1.28 ms	10.24 ms	1.25 s
TLS Handshake	300	9.37 µs	75.00 µs	9.15 ms
OTA Updates	10,240	320.00 µs	2.56 ms	312.50 ms

## Data Availability

The source code used for the experimental tests and the raw results obtained can be accessed here: https://github.com/DeepCodeWizard/Improving-IoT-Cybersecurity-Performance-with-Lifecycle-Aware-Bit-Manipulation-Compiler-Optimizations. Last accessed on 19 March 2026.
